# Supplementary material for: Cryo-EM structures of Uba7 reveal the molecular basis for ISG15 activation and E1-E2 thioester transfer
Source: Nat Commun. 2023 Aug 8;14:4786. doi: 10.1038/s41467-023-39780-z (PMC10409785; doi:10.1038/s41467-023-39780-z)
Supplement: Supplementary file 1 — Supplementary Information [file 41467_2023_39780_MOESM1_ESM.pdf]

Supplementary Information

Cryo-EM structures of Uba7 reveal the molecular basis for ISG15 activation and E1-E2 thioester transfer

Mohammad Afsar[1], GuanQun Liu[2], Lijia Jia[1], Eliza A. Ruben[1], Digant Nayak[1], Zuberwasim Sayyad[2], Priscila dos Santos Bury[1], Kristin E. Cano[1], Anindita Nayak[1], Xiang Ru Zhao[1], Ankita Shukla[1], Patrick Sung[1], Elizabeth V. Wasmuth[1], Michaela U. Gack[2], Shaun K. Olsen[1]

[1]Department of Biochemistry & Structural Biology, University of Texas Health Science Center at San Antonio, San Antonio, TX, 78229, USA

[2] Florida Research and Innovation Center, Cleveland Clinic, Port Saint Lucie, FL, 34987, USA

*Correspondence should be addressed to: olsens@uthscsa.edu

Keywords: ubiquitin-like proteins; cryo-electron microscopy; E1, E2, ISG15, conformational change, adenylation, thioester, innate immunity

Supplementary Information Inventory:

Supplementary Tables 1-2

Supplementary Figures 1-9

Supplementary Table 1 | Cryo-EM data collection, refinement, and validation statistics

	Uba7-UBE2L6-ISG15(t)/ ISG15(a) *Form 1*	Uba7-UBE2L6-ISG15(t)/ ISG15(a) *Form 2*	Uba7-UBE2L6/ ISG15(a)	Uba7-UBE2L6-ISG15(t)/ ISG15(a) composite map	Uba7-UBE2L6-ISG15(t)/ ISG15(a) consensus map
PDB ID	8SEA	8SE9	8SEB	8SV8	
EMD ID	40408	40407	40409	40782	40799
Voltage (kV)	300	300	300	300	300
Electron exposure (e-/Å^2)	80	80	53	80	80
Collection pixel size (Å)	0.8332	0.8332	0.4125	0.8332	0.8332
Initial particle images	12263879	12263879	6748350	12263879	12263879
Final particle images	76089	149051	67077	225140	225140
Symmetry imposed	C1	C1	C1	C1	C1
Resolution, unmasked, FSC threshold 0.143 (Å)	3.97	3.76	3.93	n/a	3.67
Resolution, masked, FSC threshold 0.143 (Å)	3.38	3.21	3.24	3.38	3.67
Local resolution range (Å)	2.01-5.85	2.76-5.64	2.79-6.48		
Refinement Statistics					
Refinement program	Phenix.real space	Phenix.real space	Phenix.real space	Phenix.real space	
Protein//ligand atoms(non-H)	10208/23	10195/23	9609/23	10208/23	
RMSD bond (Å)	0.003	0.003	0.002	0.012	
RMSD angles (°)	0.501	0.525	0.523	0.852	
B-factors: protein/ligand (Å^2)	118/20	107/20	287.92/20	228.62/88.83	
Ramachandran plot statistics (%)					
favored	95.97	96.43	95.48	94.42	
allowed	4.03	3.41	4.44	5.50	
Outliers	0.00	0.16	0.08	0.08	
Clash score	8.56	6.60	7.27	11	
MolProbity score	2.10	1.83	1.90	1.94	

Supplementary Table 2 | All PCR primers used in this study

Name	Primer sequence
ISG15_R92E_Fp	TAAGGGTGAAAGCAGCACCTATGAAGTGCG
ISG15_R92E_Rp	CTGCTTTCACCCTTATTATTACGAACCAGAATG
ISG15_D120R_Fp	GCAGGATCGACTGTTTTGGCTGACCTTTGAAG
ISG15_D120R_Rp	AACAGTCGATCCTGCACACCTTCCAGACC
ISG15_R155E_Fp	GCGTCTGGAGGGCGGCTAAGGTACCGAACC
ISG15_R155E_Rp	CCGCCCTCCAGACGCAGATTCATAAAAACGGTG
ISG15_Q118I_Fp	AGGTGTGATTGATGATCTGTTTTGGCTGACC
ISG15_Q118I_Rp	TCATCAATCACACCTTCCAGACCACTAACC
ISG15_N89A_Fp	TCGTAATGCCAAGGGTCGTAGCAGCACC
ISG15_N89A_Rp	CCCTTGGCATTACGAACCAGAATGCTCAGCG
ISG15_T125D_Fp	TTGGCTGGACTTTGAAGGCAAACCGCTGG
ISG15_T125D_Rp	TCAAAGTCCAGCCAAAACAGATCATCCTGC
ISG15_N151A_Fp	TTTTATGGCACTGCGTCTGCGCGGCGGCTAA
ISG15_N151A_Rp	CGCAGTGCCATAAAAACGGTGCTCAGCGGT
Ube2L6_E12K_Fp	GGAGCTGAAAGATCTTCAGAAGAAGCCTCCCCC
Ube2L6_E12K_Rp	AGATCTTTCAGCTCCTTCACCACTCGC
Ube2L6_N31A_Fp	TGATGCCGCAGTCCTGGTGTGGCACGCT
Ube2L6_N31A_Rp	AGGACTGCGGCATCATCGCTGGACAGGT
Ube2L6_K9E_Fp	AGTGGTGGAGGAGCTGGAGGATCTTCAGAAGA
Ube2L6_K9E_Rp	AGCTCCTCCACCACTCGCATGCTCGC
Uba7_E890K_Fp	TCTGGCAAAAAATTATCTGATTCGCTATATGCCGT
Uba7_E890K_Rp	TAATTTTTTGCCAGATGCAGATAACTATGACGA
Uba7_Y892A_Fp	AGAAAATGCCCTGATTCGCTATATGCCGTTTGCC
Uba7_Y892A_Rp	ATCAGGGCATTTTCTGCCAGATGCAGATAAC
Uba7_Y202A_Fp	TACCCATGCCTTTCGTGATGGTGACCTGGTGACC
Uba7_Y202A_Rp	CGAAAGGCATGGGTATTGGCACCTTTGCG
Uba7_E255K_Fp	CATTACCAAAGTGAAACGTCCGAAAACCGTTC
Uba7_E255K_Rp	TTCACTTTGGTAATGGCACCACCGCG
Uba7_D207R_Fp	TGATGGTAGGCTGGTGACCTTTAGCGGTATTG
Uba7_D207R_Rp	ACCAGCCTACCATCACGAAAATAATGGGTATTG

Uba7_H691D_Fp	GCTGCGCGACTTTCCGCCGAATAAGGTTCTGG
Uba7_H691D_Rp	GGAAAGTCGCGCAGCAGCTGCTTAATG
Uba7_R602D_Fp	TACCGTGGACTATTTTCCGAGTACCGCCGAACA
Uba7_R602D_Rp	AAATAGTCCACGGTACAAACCGGATACGGA
Uba7_R583D_Fp	AGCATATGACGCACCGGCCAGCGCAGCA
Uba7_R583D_Rp	GGTGCGTCATATGCTTCGGTCACATGCGGCAT
Uba7_E592R_Fp	AGCCAGCCGGGATGCTCCGTATCCGGTTTGTACC
Uba7_E592R_Rp	GCATCCCGGCTGGCTGCTGCGCTGGC
Uba7_K492A_Fp	TCGCCCGGCAGCAGAAGTGGCGGCAGCC
Uba7_K492A_Rp	TCTGCTGCCGGGCGACCAACATCCTGG
Uba7_D468R_Fp	CGTTGTGCGAATGGATCATATTGAACGTAGTAATC
Uba7_D468R_Rp	TCCATTCGCACAACGGTCAGACCGCC
Uba7_K810E_Fp	GTTTGAAGAAGATGATGACAGTAACTTCCATGTTG
Uba7_K810E_Rp	TCATCTTCTTCAAACATTAACGGTTTCAGCGG
Uba7_E1001K_Fp	TGACGATAAAGATACCGCATTTCCGCCGC
Uba7_E1001K_Rp	GTATCTTTATCGTCACCTTCACAGCTCAG
Uba7_D999R_Fp	TGAAGGTAGAGATGAAGATACCGCATTTCCGCC
Uba7_D999R_Rp	TCATCTCTACCTTCACAGCTCAGTTCCAGC

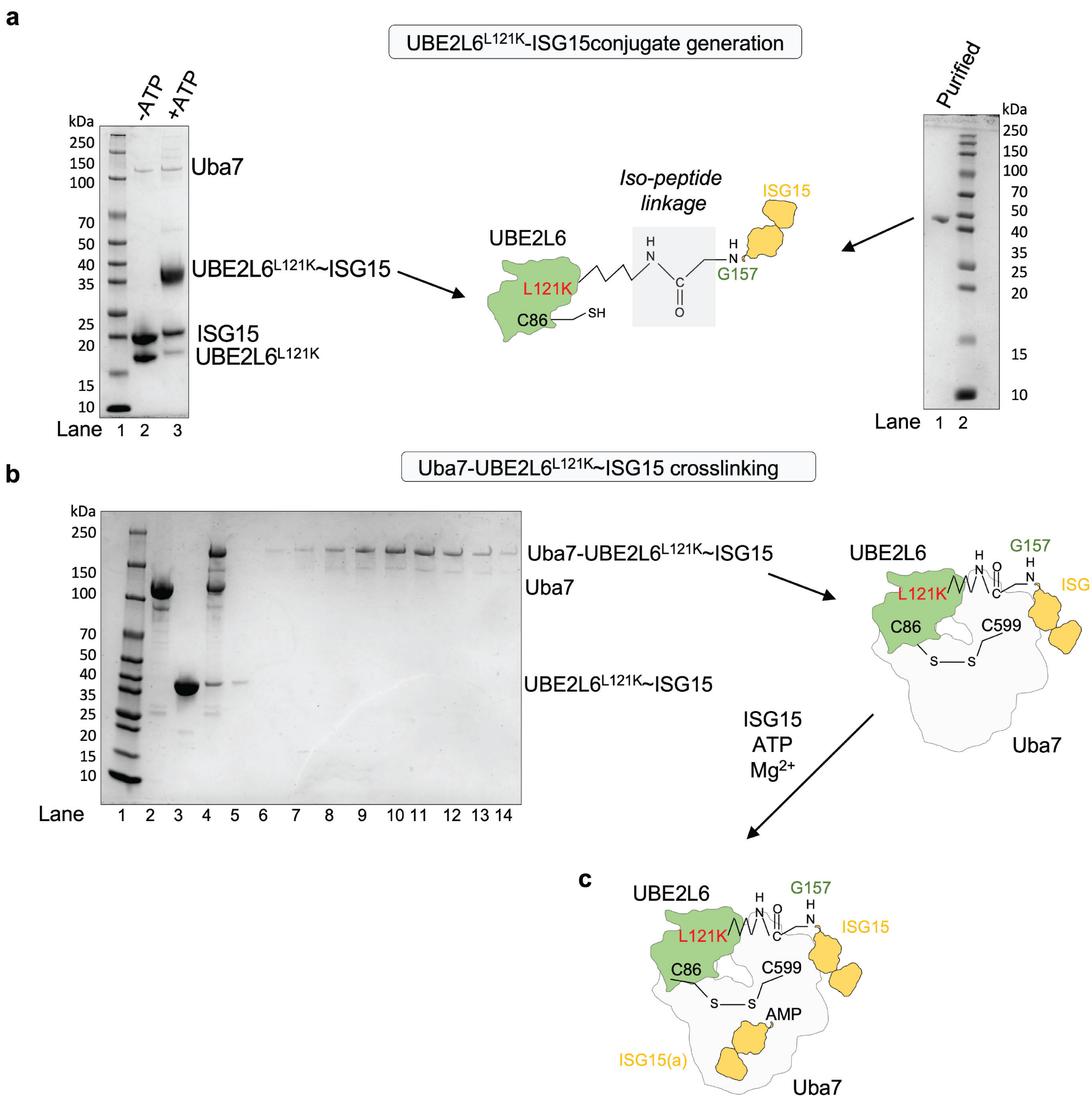

Supplementary Fig. 1 | Trapping a double-loaded Uba7-UBE2L6-ISG15(t)/ISG15(a) complex

a, Purification UBE2L6^{L121K}-ISG15(t) conjugate **b,** Chemical crosslinking of active site cysteines and purification of Uba7-UBE2L6^{L121K}~ISG15(t) complex. For making doubly-loaded, complex we incubated Uba7-UBE2L6^{L121K}~ISG15(t) complex with ISG15 ATP and Mg and allowed to adenylate the ISG15 in AAD domain to form Uba7~ISG15(a)-UBE2L6^{L121K}~ISG15(t).

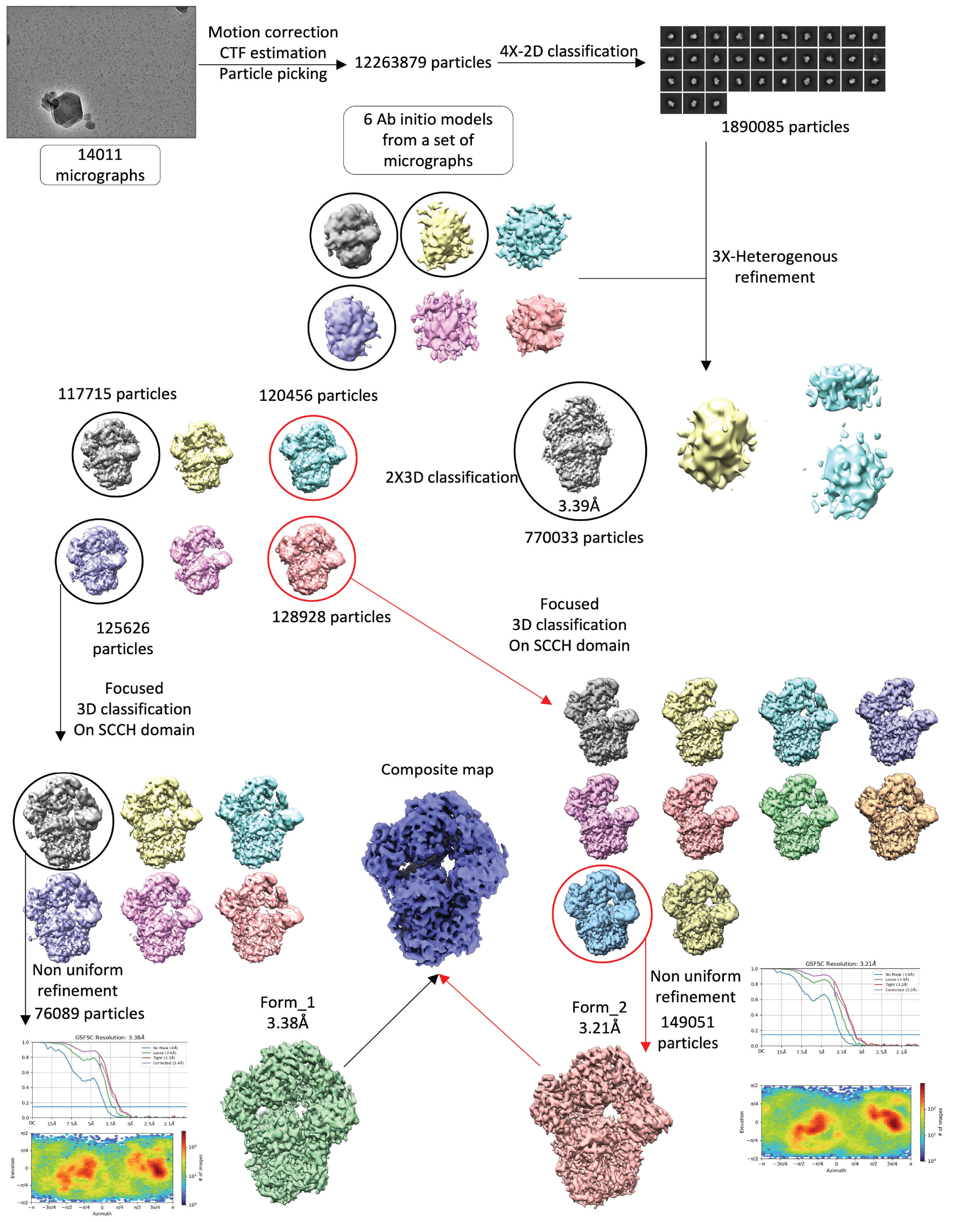

Supplementary Fig. 2 | Cryo-EM data processing of the double-loaded Uba7-UBE2L6-ISG15(t)/ISG15(a)

Cryo EM workflow depicting major steps of 2D and 3D classification with final masked FSC curveand orientational distribution of final map.

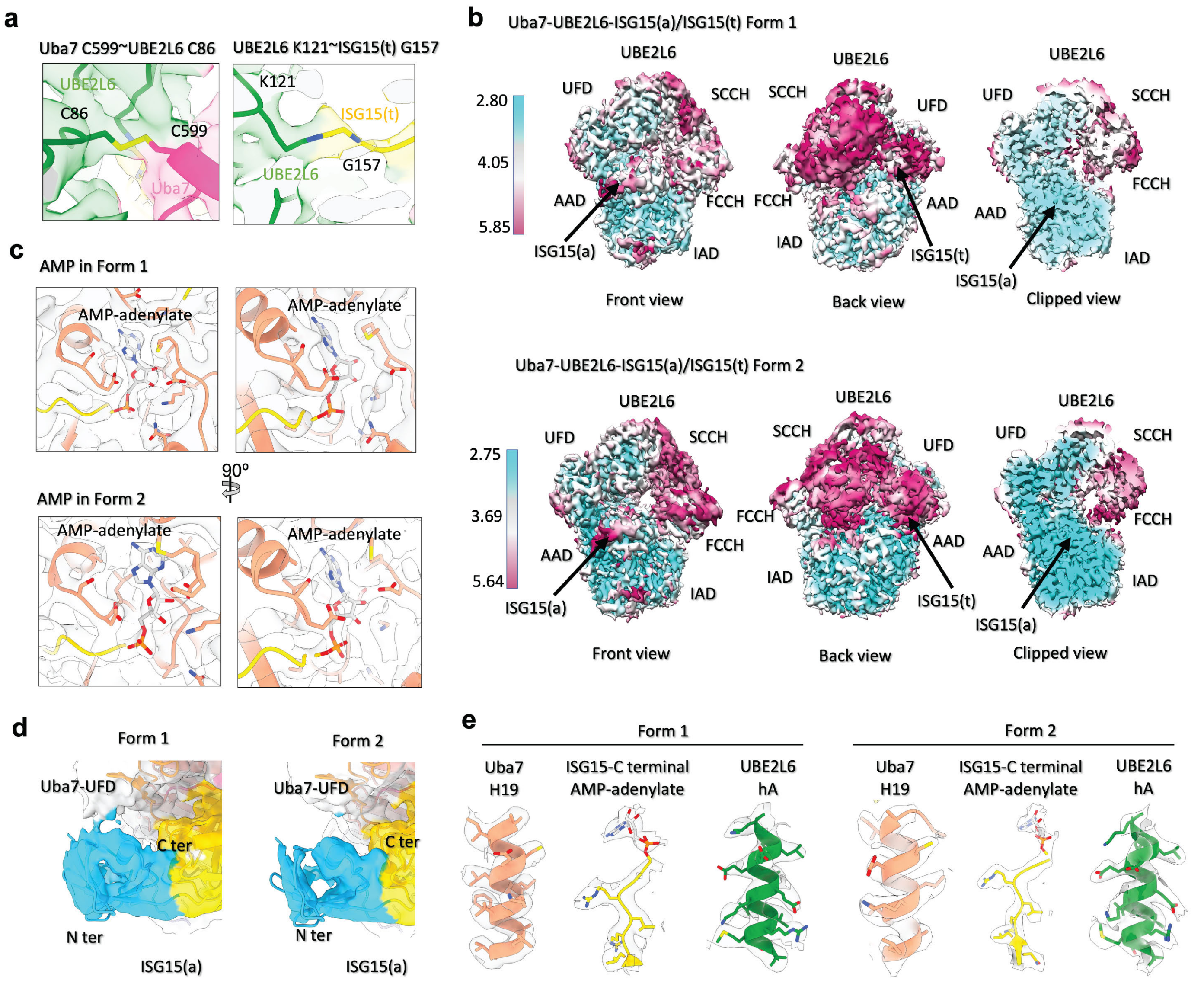

Supplementary Fig. 3 | Quality of the double-loaded Uba7-UBE2L6-ISG15(t)/ISG15(a) reconstruction
a, Cryo-EM density map showing the disulfide bond between the active site cysteines of Uba7 (C599) and UBE2L6 (C86) in the *left panel*, and the isopeptide bond between the UBE2L6 (K121) and ISG15(t) (G157) in the *right panel*. **b**, Local resolution estimation and visualization of Uba7-UBE2L6-ISG15(t)/ISG15(a) Form 1 (top) and Form 2 (bottom) **c**, Cryo-EM density map for AMP-Gly157-ISG15(a) for Forms 1 and 2. **d**, The density of Form 1 and Form 2, showing the density for N terminal domain of ISG15 at lower threshold. **e,** The density map of Form1 and Form 2, showing the key structural features of Uba7 H19, ISG15 C terminal with AMP and UBE2L6 hA.

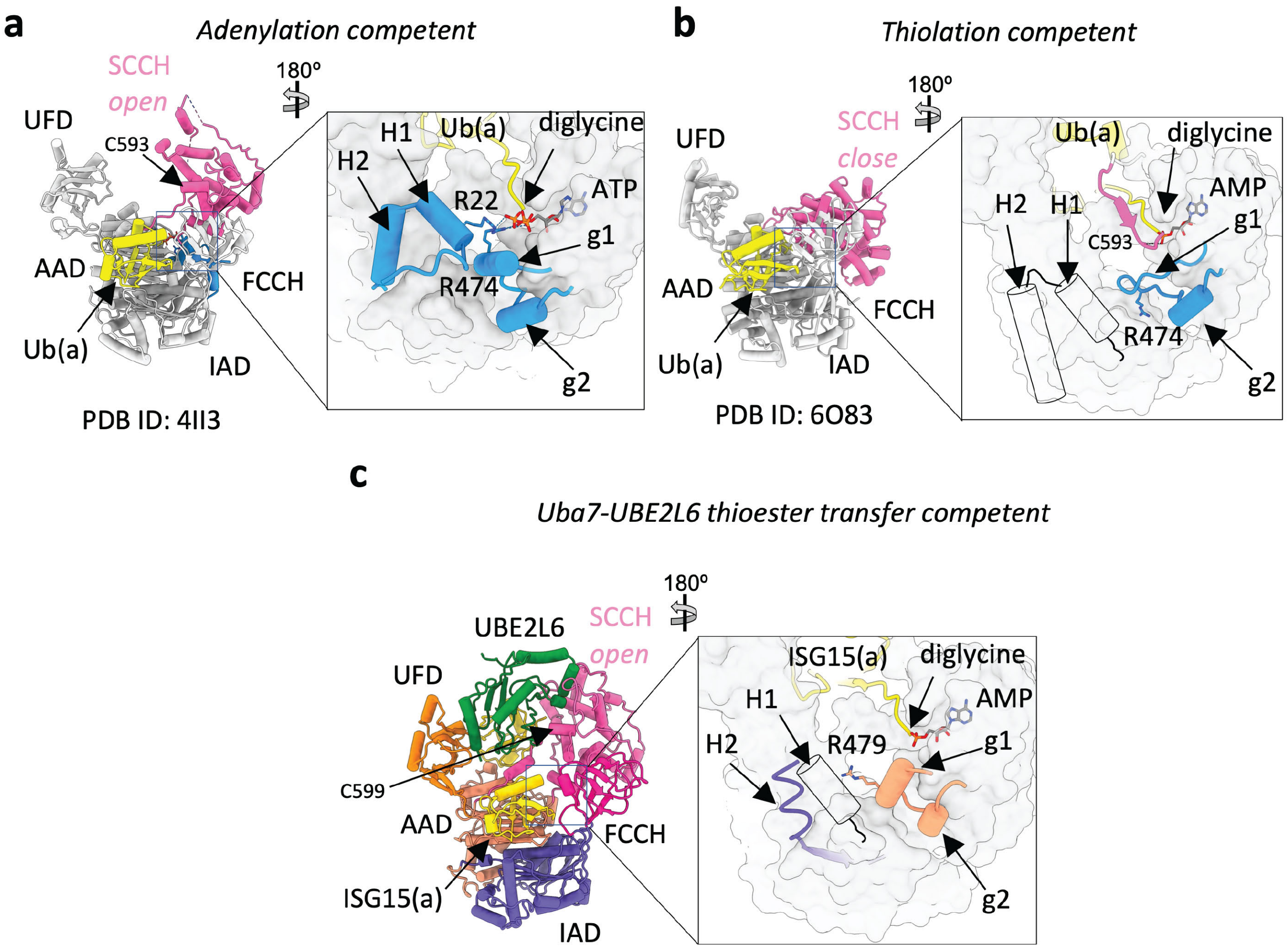

Supplementary Fig. 4 | Active site remodeling during thioester formation and transfer

a, The adenylation-competent structure of Uba1/Ub (PDB: 4II3) illustrates the positioning of catalytically important H1/H2 and g1/g2 helices in the active site. R474 (from g1 helix) and R22 (from H1 helix) participate in hydrogen bonds with b-and g-phosphates of ATP. **b,** the thiolation-competent structure of Uba1/Ub (PDB: 6O83) illustrates disordering of the H1/2 and g1 helices in the active site, which effectively disassembles catalytic machinery for adenylation. Note that residues important for adenylation are drastically changed including R22 from H1/H2, which is disordered and R474 which extends away from the active site. This contrasts the proximity of these residues to the b-and g-phosphates of ATP in the adenylation-competent structure shown in panel a. **c,** The Uba7-UBE2L6 thioester transfer-competent structure harbors ordered g1/g2 helices as in the adenylation-competent structures but disordered H1/H2 region as in thiolation-competent structures. We speculate that disorder of the H1/H2 region partially accounts for the conformational dynamism of the SCCH domain in our Uba7-UBE2L6-ISG15(t)/ISG15(a) structure, as the SCCH domain is typically observed perched atop the H1/H2 helices when in the open, adenylation-competent conformation.

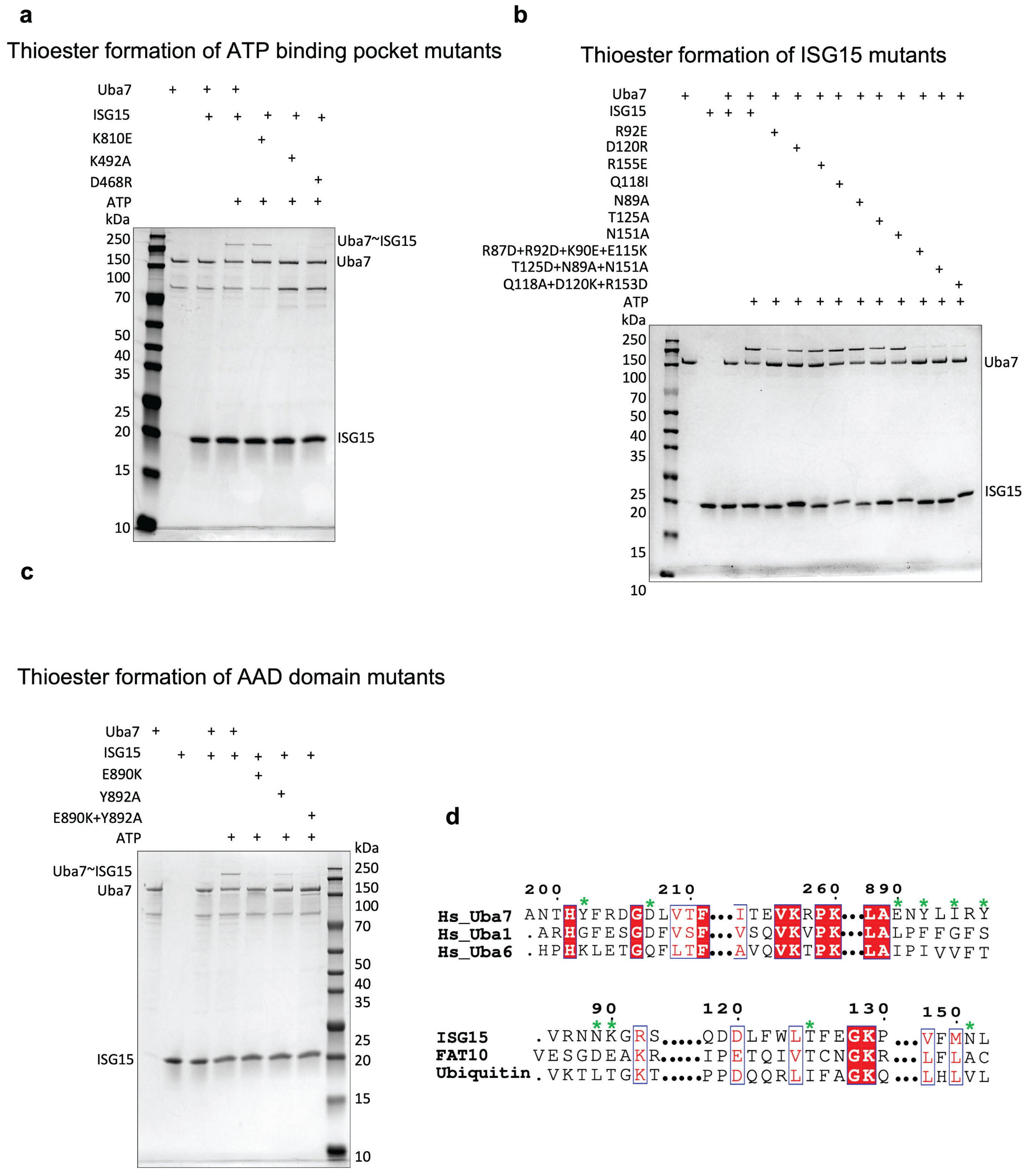

Supplementary Fig. 5 | Structure-function analysis of the Uba7/ISG15(a) interface

a, Uba7~ISG15 thioester formation activity of Uba7 active site mutants. **b,** Uba7~ISG15 thioester formation activity of ISG15(a) mutants. **c,** Uba7~ISG15 thioester formation activity of Uba7 AAD mutants. **d,** Multiple sequence alignment of Uba7, Uba1 and Uba6. Residues which contact ISG15(a) are highlighted with green asterisks. Source data for **a-c** are provided as source data file for all three technical replicates.

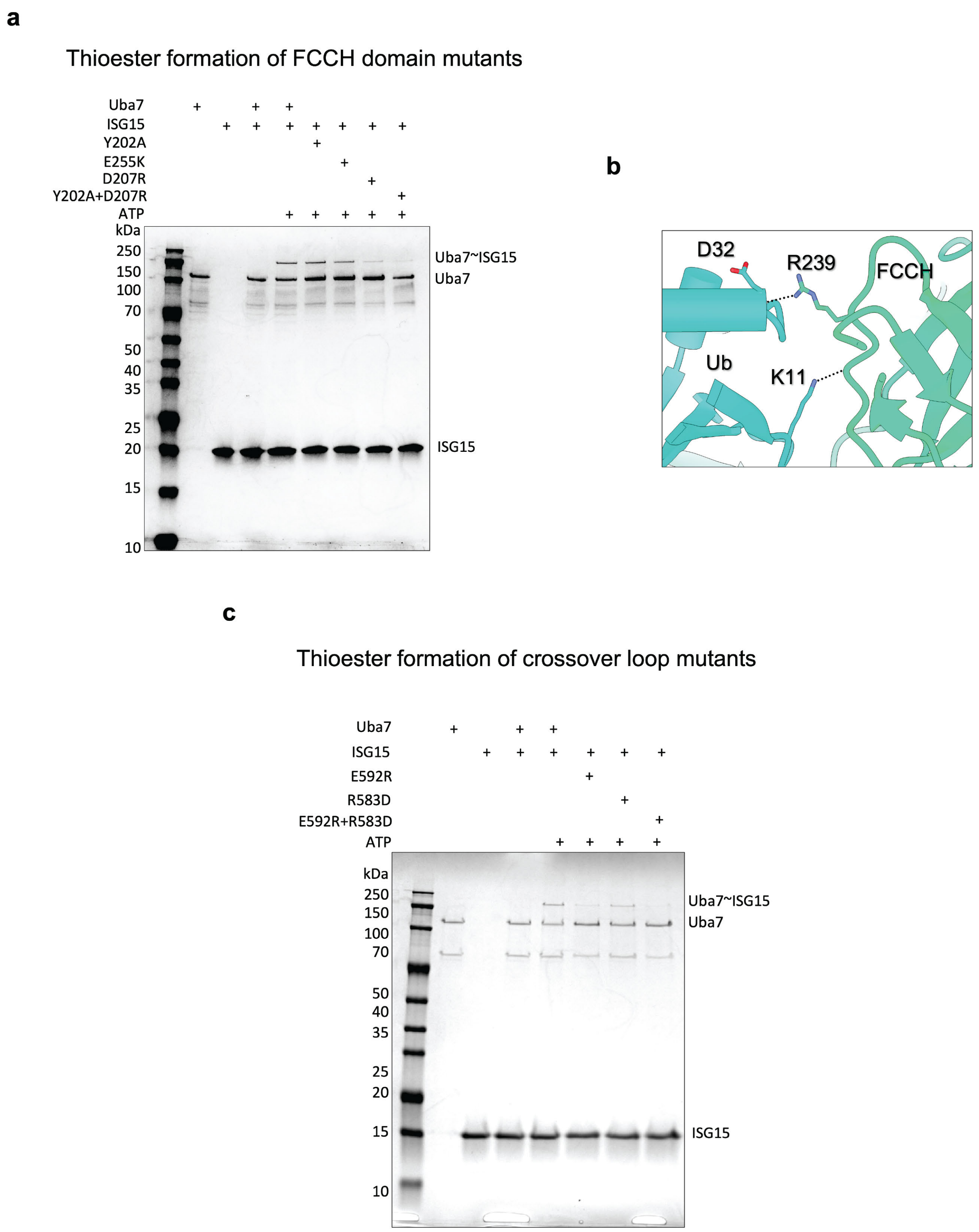

Supplementary Fig. 6 | Structure-function analysis of the Uba7 FCCH and crossover loop/ISG15(a) interfaces

a, Uba7~ISG15 thioester formation activity of Uba7 FCCH domain mutants. **b,** Interaction interface between the Ub(a) and FCCH domain of Uba1. **c,** Uba7~ISG15 thioester formation activity of Uba7 crossover loop mutants which interact with ISG15(a). Source data for **a** and **c** are provided as source data file for all three technical replicates.

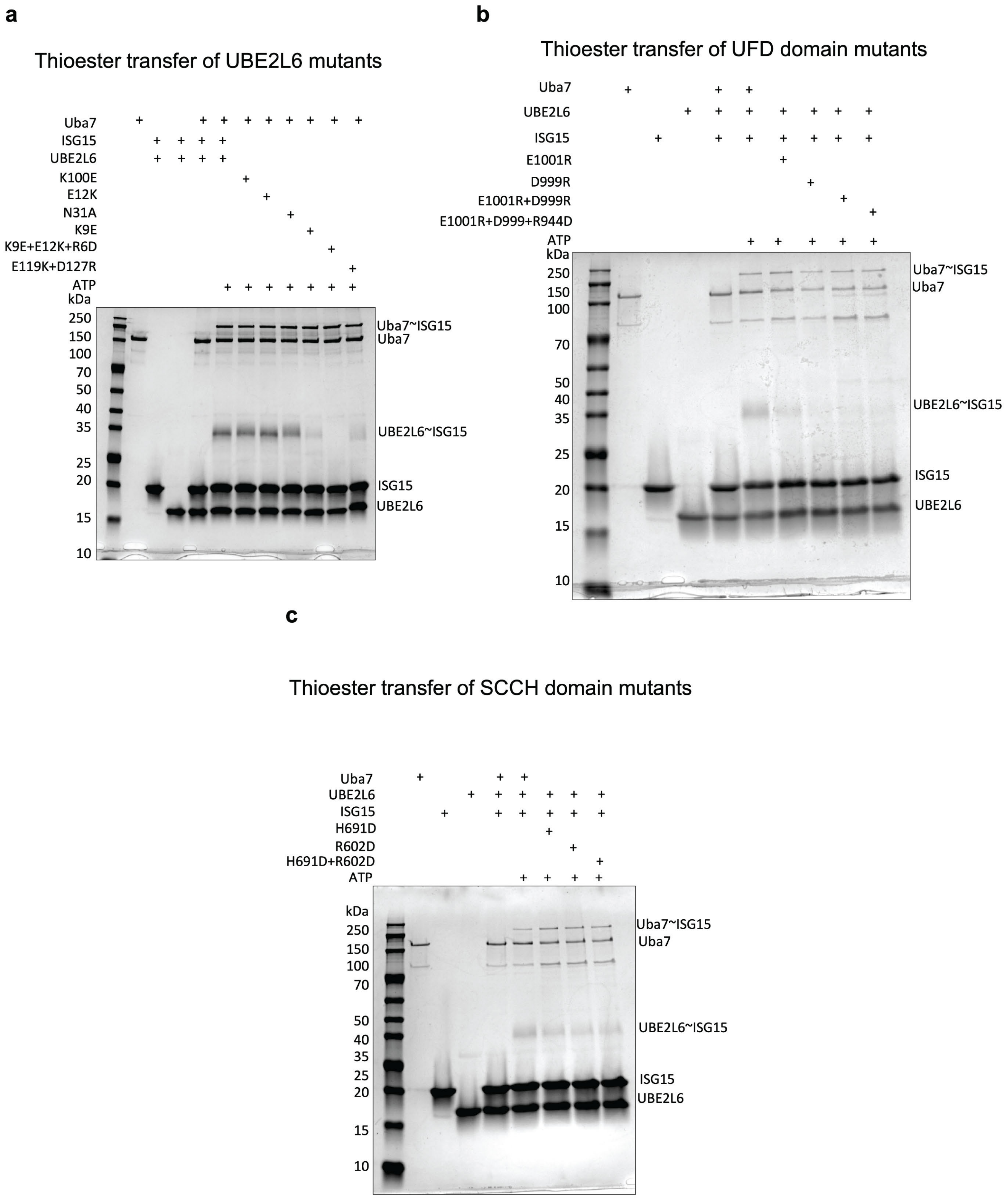

Supplementary Fig. 7 | Structure-function analyis of the Uba7/UBE2L6 interface

a, Thioester transfer activity of UBE2L6 mutants which interacts with UFD and SCCH domain of Uba7. **b,** Thioester transfer activity of UFD domain mutants which interacts with hA of UBE2L6. **c,** Thioester transfer activity of SCCH domain mutants which interacts with hC of UBE2L6. Source data for **a-c** are provided as a source data file for all three technical replicates.

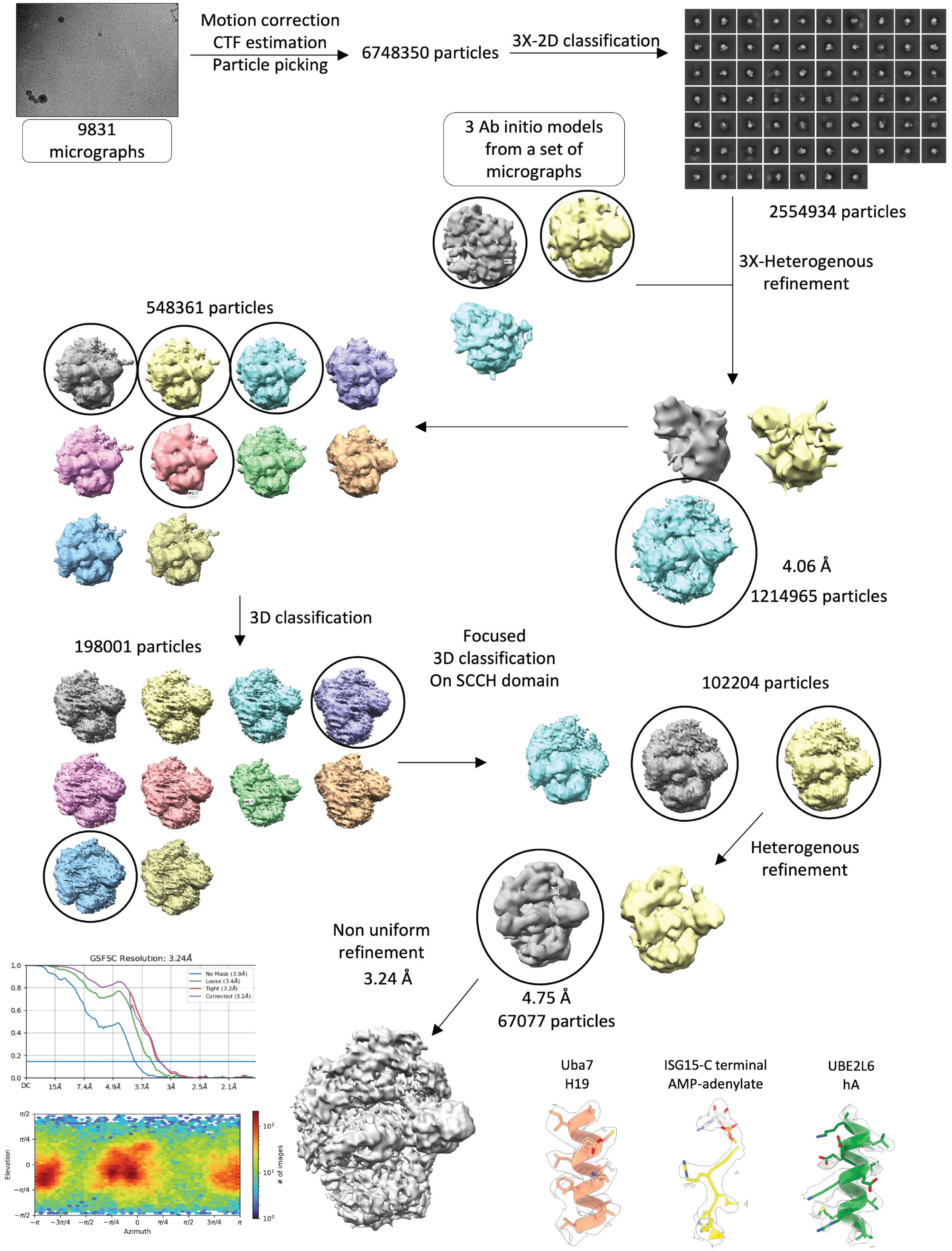

Supplementary Fig. 8 | Cryo-EM data processing of the single-loaded Uba7-UBE2L6/ISG15(a) complex

Cryo-EM data processing workflow depicting major steps of 2D and 3D classification with final masked FSC curves and orientation distribution of the final map. The density map showing the key structural features of Uba7 H19, ISG15 C terminal with AMP and UBE2L6 hA are also shown.

a

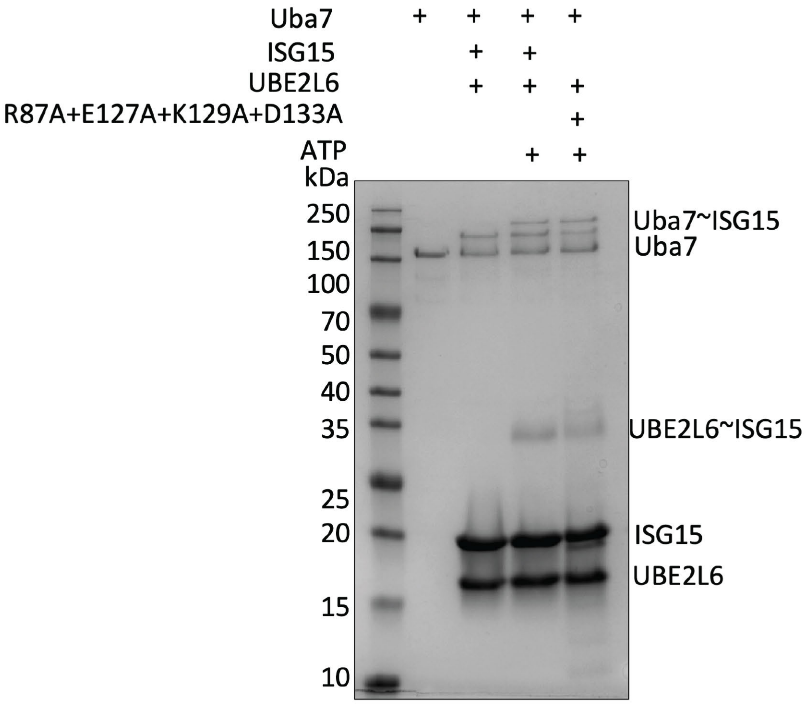

Supplementary Fig. 9 | Structure-function analysis of the Uba7/ISG15(t) interface

a, Uba7-UBE2L6-ISG15 thioester transfer activity of the indicated ISG15 mutant. Source data are provided as a source data file for all three technical replicates.
