# Supplementary material for: Cryo-EM structures of Uba7 reveal the molecular basis for ISG15 activation and E1-E2 thioester transfer
Source: Nat Commun. 2023 Aug 8;14:4786. doi: 10.1038/s41467-023-39780-z (PMC10409785; doi:10.1038/s41467-023-39780-z)
Supplement: Supplementary file 4 — Description of Additional Supplementary files [file 41467_2023_39780_MOESM4_ESM.docx]

*Descriptions of Additional Supplementary files*

**Supplementary Movie 1 | 3D variability analysis of Uba7-UBE2L6-ISG15(t)/ISG15(a) complex**

The 3D variability analysis of Uba7-UBE2L6-ISG15(t)/ISG15(a) complex shows crosstalk between the NTD of ISG15(a) with UFD domain of Uba7 at low resolutions.

**Supplementary Movie 2 | Conformational changes accompanying ISG15 activation and thioester transfer by Uba7**

The movie provides a model of how Uba7 catalyzes ISG15 adenylation, thiolation, and E1-E2 thioester transfer to UBE2L6.
